# Cognitive, language, and school performance in children and young adults treated for low‐grade astrocytoma in the posterior fossa in childhood

**DOI:** 10.1002/cnr2.1494

**Published:** 2021-07-07

**Authors:** Ingela Kristiansen, Cristina Eklund, Margareta Strinnholm, Bo Strömberg, Maria Törnhage, Per Frisk

**Affiliations:** ^1^ Department of Women's and Children's Health Uppsala University and Uppsala University Children's Hospital Uppsala Sweden

**Keywords:** childhood, cognitive outcome, language and learning difficulties, pilocytic astrocytoma, posterior fossa

## Abstract

**Background:**

Pilocytic astrocytoma is the most common brain tumour type in childhood located in the posterior fossa, and treated mainly with surgery. These tumours have low mortality, but knowledge concerning its long‐term outcome is sparse.

**Aim:**

The aim of this study was to investigate whether children treated for pilocytic astrocytoma in the posterior fossa had late complications affecting cognition, language and learning.

**Methods:**

This descriptive single‐centre study includes eight children and 12 adults treated as children for pilocytic astrocytoma in the posterior fossa, with a mean follow‐up time of 12.4 (range 5–19) years. Well‐established tests of intelligence, executive, language and academic function were used.

**Results:**

Intelligence tests showed average results compared with norms. Five patients scored <−1 SD (70–84) and 3 low average (85–92) on full scale IQ. The patients scored average on subtests regarding executive function, except for significantly lower results in inhibition/switching (p = .004). In Rey complex figure test half of the patients scored below −1 SD. Language tests were normal except for significantly lower results in naming ability (p = .049) and in inference (p = .046).

In academic tests, results were average, except for significantly lower results in reading speed (p = .024). Patients with learning difficulties performed worse in the tests.

**Conclusions:**

The patients' functional outcome was favourable but, a not‐negligible part of the patients displayed neurocognitive difficulties as revealed by extensive neuro‐cognitive and academic testing. Thus, it is important to identify those in need of more thorough cognitive and pedagogic follow‐up programmes, including school interventions.

## 1 INTRODUCTION

The yearly incidence of brain tumours in Sweden during 1984–2005 was 4.2/100 000 in children younger than 15 years. Survival rates have improved but vary across different tumour types.^1^ There are also variations regarding medical, cognitive, and psychological long‐term outcome. This may be caused by the tumour itself or by the treatment.^2^ Long‐term consequences are mainly reported among children treated for high‐grade tumours,^3^ but in an earlier study, where all children treated for brain tumours at Uppsala University Children's hospital 1995–2006 were studied, we found physical and cognitive complications also among children treated for low‐grade tumours.^4^ There is controversy regarding the role of tumour location. Studies have demonstrated that supratentorial tumours entail higher morbidity than infratentorial tumours.^5^ In a study by Patel,^6^ the supratentorial and infratentorial tumour location groups did not differ regarding intellectual functioning. However, survivors of infratentorial tumours performed worse on selected measures of intellectual functioning and on parent‐reported social–emotional functioning.

The cerebellum has an important role in coordinating different neurological functions. Injuries may affect executive function, spatial cognition, personality, and language,^7^, ^8^, ^9^ as well as fine‐^10^ and gross‐motor performance.^11^ A cerebellar cognitive affective syndrome (CCAS) has been described, characterised by deficits in executive function, spatial cognition, linguistic processing, and affect regulation.^7^, ^9^ The post‐operative paediatric cerebellar mutism syndrome (pCMS), defined in a consensus paper by Gudrunadottir is another known complication after posterior fossa surgery.^12^ It typically manifests 1–2 days after surgery and affects speech, emotions, personality, and behaviour.^13^ The mutism is always transient, but recovery may be prolonged.^12^ pCMS affects mainly children treated for medulloblastoma but may also occur in children treated for ependymoma or pilocytic astrocytoma.^13^, ^14^

Pilocytic astrocytoma is the most common brain tumour type in childhood located in the posterior fossa, mainly treated with surgery.^5^, ^15^ These tumours have a low mortality and the long‐term functional outcome is favourable.^5^ Although there are studies describing neurological, cognitive, and behavioural complications among these patients, there is still a lack of knowledge concerning the long‐term outcome.^16^, ^17^, ^18^, ^19^, ^20^, ^21^, ^22^ In one study, Traunwieser reported that all patients treated for low‐grade glioma (LGG) were at risk of experiencing long‐term cognitive impairments. These deficits occurred even following complete/subtotal resection of cerebellar LGGs.^23^ In earlier studies, we found a favourable clinical outcome, but some patients reported neurological complications and learning difficulties which were unmet in school.^4^, ^24^

Against this background of ambiguous results of long‐term functional outcome for children treated for low‐grade pilocytic astrocytoma in the posterior fossa, our aim was to investigate whether these patients had complications affecting intelligence, executive function, memory, language and academic performance and if some fulfilled the criteria for CCAS or suffered from pCMS. We also wanted to investigate if the patients had learning difficulties and if this had led to increased educational support.

## 2 MATERIALS AND METHODS

### 2.1 Participants

This single‐center study was performed 2015–2017 at Uppsala University Children's Hospital, Sweden, a tertiary referral center for children with tumours in the central nervous system, serving six counties in mid‐Sweden with a population of 1.7 million people. Patients were retrieved from the local and the National Brain Tumour Registry. A total of 27 patients <18 years of age with a low‐grade astrocytoma in the posterior fossa diagnosed and treated in childhood 1995 through 2011 were identified. At the time of this investigation, nine were still children (9–17 years) and 18 young adults (21–33 years). All patients had at least 5 years of follow‐up after the end of treatment.

This study includes the same patient group as a former study from our research group^24^ except two adults who only participated in telephone interviews. Three patients did not answer several invitations, and two declined participation. Thus, 20 patients agreed to take part (12 adults and eight children; 74%). The mean age at tumour presentation was 8.3 years (SD 4.3), and the mean age at participation in the study was 20.2 (SD 7.3) years, range 9–33 years. Mean time from diagnosis to participation was 12.2 (SD 4.6) years.

All patients were treated surgically, and in 17 patients the operation was considered as a complete resection. Three patients had a remaining tumour and were re‐operated shortly after the initial operation. Another three patients relapsed; one was treated with re‐surgery, one with re‐surgery and chemotherapy and one with gamma knife radio surgery. These three patients did not differ from the rest although the patient treated with only re‐surgery reported learning difficulties and scored low average in full‐scale intelligence quotient (FSIQ).

### 2.2 Methods

Upon acceptance, participants were asked to come to the Folke Bernadotte Regional Rehabilitation Centre to undergo investigations performed by a multi‐professional team, 2 days for adults and 3 days for children. The investigations included an interview, a neurological examination, neuropsychological investigations, tests of language and, for children, screening tools for academic performance. The tests were chosen based on which type of difficulties that are common in patients treated for conditions affecting the cerebellum and on local clinical experience of these specific tests.

### 2.3 Neuropsychological tests

#### 2.3.1 Intelligence tests

Swedish versions of the intelligence tests, Wechsler Intelligence Scale for children 6–16 years (WISC‐IV and ‐V)^25^, ^26^ and for adults (>16 years), Wechsler Adult Intelligence Scale (WAIS‐IV)^27^ were used. During the time of the investigations WISC‐V was introduced and used clinically at our institution. WISC‐IV and WAIS‐IV include four primary index scales: verbal comprehension, perceptual reasoning, working memory and processing speed. WISC‐V includes five primary index scales: verbal comprehension, visual spatial, fluid reasoning, working memory, and processing speed. It is also possible to calculate FSIQ. Results were calculated into index scores, mean (M) 100, and standard deviation (SD) 15.

#### 2.3.2 Tests of executive function

*Parts of Delis–Kaplan executive function system (D‐KEFS*)^28^ were used: Trail making test (TMT), number‐letter switching, which assesses flexibility of thinking on a visual‐motor sequencing task. The Tower test measures spatial planning, rule learning, inhibition of impulses, perseverative responding, and the ability to establish and maintain the instructional set. The Color‐Word Interference Test (Colour‐word inhibition and Colour‐word inhibition/switching) evaluates inhibition and cognitive flexibility. Results were calculated in scaled scores (M10, SD 3) using American norms.

*Behaviour Rating inventory of executive function (BRIEF)* is a standardised questionnaire for assessing executive functions in children, adolescents, and adults.^29^ There are separate questionnaires for patients and their parents/relatives. Each questionnaire consists of 86 items divided into eight clinical scales: inhibit, shift, emotional control, monitor, working memory, plan/organise, organisation of materials, and task completion. It yields a Global executive composite score (total score, GCS), a behavioural regulation index (BRI), and a Metacognition Index (MI). Results were calculated into T‐scores (M 50, SD 10) using American norms. T‐scores at or above 65 indicate clinically significant executive difficulties.

#### 2.3.3 Test of visuospatial construction skills and visuospatial memory

*Rey complex figure test* (*RCT*) was used to asses visuospatial construction and memory,^30^ using two parts of the test, immediate and delayed recall. Results were calculated into T‐scores (M 50, SD 10) with American norms.

### 2.4 Tests of language functions

*Clinical evaluation of language fundamentals, fourth edition (CELF‐4)* ^31^ is an instrument for identifying language difficulties and disorders in children 5–12:11 years. Five indices are included in this study: Core language score (general language ability), receptive language, expressive language, language content, and language memory. Results were calculated into index scores (M 100, SD 15) with Scandinavian norms.

*Boston naming test (BNT)* is a test of naming ability, with Swedish normative data for the ages 21–80 years (M 53.04, SD 4.18).^32^

*Assessment of subtle language disorders (BeSS‐ Bedömning av Subtila Språkstörningar)* is a Swedish test of high‐level language functions.^33^ It consists of seven parts: repetition of long sentences, construction of sentences, inference, comprehension of logico‐grammatical sentences, comprehension of ambiguous sentences, understanding of metaphors, and definition of words. Maximum level on each test is 30 points. Normative Swedish data for each item are based on an investigation of 28 high school students (16–19 years)^33^; the levels of M and SD differ across the subtests.

*Tests of word fluency (FAS, animal and verb fluency)* measure the subject's ability to generate words within a restricted time limit and within a certain category. In this study, the letter (or phonemic) fluency test called FAS (using the initial letters F, A, and S) was used^34^ together with a semantic (or category) fluency test aimed at semantic categories (animals) and actions (verbs).^34^ Swedish norms were used^34^ for the ages 16–29 years: Length of education ≤12 years FAS M 37.5 SD 11.4, animal fluency M 23 SD 4.0 and verb fluency M 17 SD 3.7; length of education >12 years M 42.4, SD 10, animal fluency M 26.4, SD 5.1 and verb fluency M 23 SD 6.3.

### 2.5 Tests of academic performance

*Diagnostic manual for analysis of reading and writing skills* is a screening instrument for assessing spelling, word knowledge, reading velocity, and reading comprehension.^35^ Swedish normative data exist for children aged 7–17 years.

*Adler screening tools for mathematical skills* is a test of mathematic performance in arithmetic for children 7–19 years.^36^ All results were converted into the stanine scale where the normal distribution is divided into nine steps (M 5, SD 1.97).

## 3 STATISTICS

Statistics were calculated using the SPSS statistical program, version 26. One sample Wilcoxon signed rank test was used due to the small sample size and unknown distribution. Spearman's rank correlation coefficient was calculated to investigate the association of age at diagnosis with neurocognitive, language, and academic performance. The level of significance was set at p < .05. All analyses were exploratory and nominal significance levels are reported without adjustment for multiplicity.

## 4 RESULTS

### 4.1 Neuropsychological tests

#### 4.1.1 Intelligence tests

Four children and 12 adults performed WISC‐IV and WAIS‐IV and four children performed WISC‐V except for verbal comprehension, where one child did not complete the subtest. Results are summarised in Table 1. In WISC‐IV and WAIS‐IV, FSIQ show a mean of 97.8 (SD 17.0). Mean results in verbal comprehension were 93.1 (SD 16.2), in perceptual reasoning 107.1 (SD 19.0), in working memory 97.6 (SD 14.5) and in processing speed 94.3 (SD 11.6). In FSIQ 3 scored low average (IQ 85–92) and 4 borderline (IQ 70–84).

**TABLE 1:** Results of WISC‐IV, WISC‐V and WAIS‐IV (reference M 100, SD 15)

	Number	Mean	SD	Median	Minimum	Maximum	p‐values
WISC‐IV and WAIS‐IV							
FSIQ	16	97.8	17.0	98.5	72	122	0.609
Verbal comprehension	16	93.1	16.2	95.0	67	124	0.101
Perceptual reasoning	16	107.1	19.0	111.0	68	134	0.126
Working memory	16	97.6	14.5	93.0	76	122	0.500
Processing speed	16	94.3	11.6	94.0	76	117	0.061
WISC V							
FSIQ	4	96.5	11.8	101.5	79	104	1.000
Verbal comprehension	3	86.7	10.3	84.0	78	98	0.109
Visuospatial index	4	98.5	11.7	101.0	84	108	1.000
Fluid index	4	97.0	10.7	100.0	82	106	0.854
Working memory	4	109.3	9.9	107.5	100	122	0.109
Processing speed	4	106.5	19.1	107.0	83	129	0.465

In WISC‐V mean result for FSIQ was 96.5 (SD 11.8). Mean results in verbal comprehension were 86.7 (SD 10.3), in visuospatial index 98.5 (SD 11.7), in fluid index 97.0 (SD 10.7), in working memory 109.3 (SD 9.9), and in processing speed 106.5 (SD 19.1). There was no correlation between age at diagnosis and results of WISC‐IV and WAIS‐IV (p = .154) or WISC‐V (p = .895).

#### 4.1.2 Tests of executive function

**FIGURE 1:**
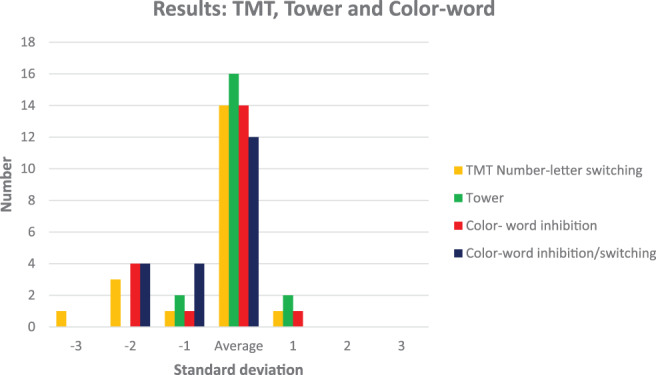
Results of tests of executive functions (TMT, Tower and Colour‐word)

Results for the subtests of D‐KEFS (Figure 1) show mean results for TMT number‐letter switching, 8.6 (SD 4.5) and for D‐KEFS tower 10.4 (SD 2.8). In colour‐word inhibition mean results were 8.1 (SD 4.0) and in color‐word inhibition/switching 6.9 (SD 3.9). The result for color‐word inhibition/switching was significantly below the norm, p = .004.

In BRIEF, patients scored mean 46.3 (SD 6.1) in GCS and parents/relatives 44.3 (SD 5.1). In BRI patients scored 44.7 (SD 5.3) and parents/relatives 43.9 (SD 5.8) and in MI patients scored mean 47.7 (SD 8.1) and parents/relatives 45.9 (SD 6.3). The results were below the cut‐off level for executive difficulties.

#### 4.1.3 Test of visuospatial construction skills and visuospatial memory

**FIGURE 2:**
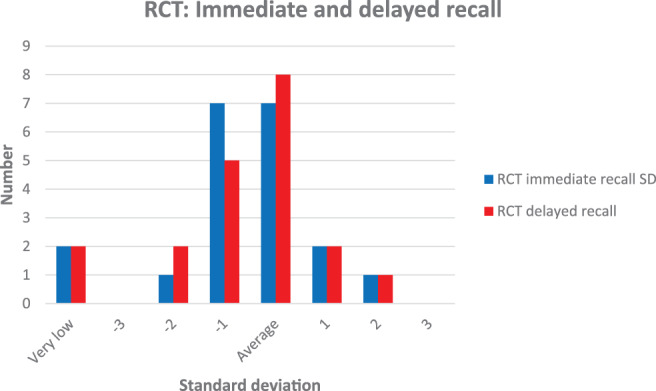
Results of Rey complex figure test: Immediate and delayed recall

All patients performed RCT, but two children had very low results on both subtests that did not yield any valid T‐scores and were therefore not included when the mean scores for the sample were calculated. For immediate recall the mean value was 47.0 (SD 14.4) and for delayed recall 45.8 (SD 13.9). The results were in the normal range, with a distribution showed in Figure 2. However, 10 patients had results below average, including those with very low results on immediate recall and nine on delayed recall.

### 4.2 Tests of language function

Five children between 9 and 13 years completed the tests in CELF‐4. Results for the core language skills show a mean value of 106.6 (SD 15.5). For receptive language the mean value was 109.2 (SD 15.6), expressive language 107.4 (SD 13.4), language content 112 (SD 14.0), and language memory 101 (SD 16.2). All results were in the normal range.

Sixteen patients performed BNT (13–33 years), the mean value was 49.8 (SD 7.1), which was significantly below the norm (p = .049). Two adult participants had results <−2 SD; 2 adults and 1 child aged 14 had a result below <−1 SD.

Fifteen patients (aged 14–33 years) performed BeSS. Mean results for repetition of long sentences was 22.3 (SD 4.0), construction of sentences 26.5 (SD 3.5), inference 22.0 (SD 5.6), comprehension of logico‐grammatical sentences 27.2 (SD 2.9), comprehension of ambiguous sentences 26.9 (SD 2.8), understanding of metaphors 24.9 (SD 4.8), and definition of words 23.9 (SD 4.6). The results were not significantly different from mean, except for inference, where results were significantly below mean (p = .046) and comprehension of logic‐grammatic sentences (p = 0.01) and comprehension of ambiguous sentences (p = 0.001), which were significantly above mean.

Sixteen patients performed FAS (aged 13–33 years), 11 had a length of education ≤12 years and five had ≥12 years. Mean results for FAS for patients with a length of education ≤12 years was 34.2 (SD 7.4), for animal fluency 20.6 (SD 4.6) and for verb fluency 17.4 (SD 3.6). The participants with a length of education of >12 years scored mean results for FAS of 41.2 (SD 10.2), for animal fluency 26.6 (SD 4.3) and for verb fluency 19 (SD 7.1). All results were within the norms.

### 4.3 Tests of academic performance

**FIGURE 3:**
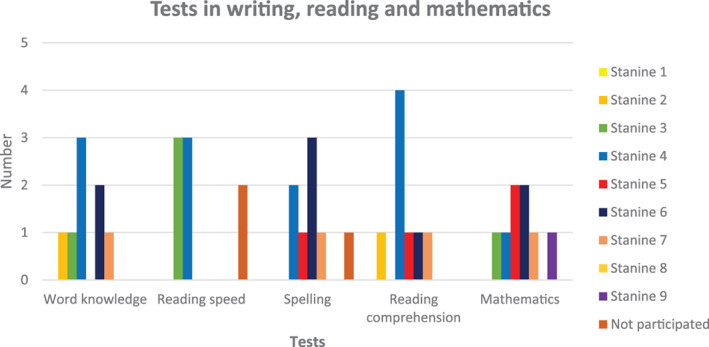
Results of the academic tests in writing, reading, and mathematics

Eight children completed the tests; two children did not perform the reading speed test due to low age, and one patient did not perform the spelling test. Mean results in word knowledge were stanine 4.5 (SD 1.7), reading speed 3.5 (SD 0.5), spelling 5.4 (SD 1.1), reading comprehension 4.5 (SD 1.5) and mathematical skills 5.6 (SD 1.8) (Figure 3). Results for reading speed were significantly lower compared with norms (p = .024), and the other items were comparable with norms. There was a significant correlation between age at diagnosis and reading speed (p = .017).

## 5 DISCUSSION

In the present study we have investigated cognitive, language, and academic performance in a group of children and young adults treated for pilocytic astrocytoma in the posterior fossa. The results of the intelligence tests show that the patients scored average results in all indices with a scattered pattern. In a study by Khajuria^37^ on different types of cerebellar tumours tested with WISC‐III, patients treated for pilocytic astrocytoma all scored low regarding performance IQ.This is not in coherence with our findings, where patients scored the highest results in perceptual reasoning, the subtest most resembling performance. One explanation for this can be that perceptual reason in later versions of WISC and WAIS include different indices and subtests compared with WISC‐III, with less demands on motor skills and processing speed.^27^ Thus, the different versions are not entirely comparable and may have affected the result.

In the tests of executive functioning, four had results <−2 SD and 1 < −1 SD in colour‐word inhibition, and the result for inhibition/switching was significantly below average. This indicates difficulties with inhibition and cognitive flexibility, which are parts of the executive processes responsible for purposeful goal‐directed behaviour and the ability to perform novel or complex tasks.^38^ Even if the mean results in RCT was in the normal range, 50%–45% of the patients had results < −1 SD in immediate and delayed recall respectively, including two children with very low results. This can indicate difficulties with visuospatial construction and memory. In addition, RCT can provide information about executive performance, for example cognitive flexibility, organisation, and working memory.^39^ Thus, our findings suggest impaired executive functioning. This is in line with findings reported by Koustenis who found that children treated for both low‐ and high‐grade tumours in the cerebellum exhibited similar patterns of impairment in executive functioning. Although deficits were larger in those treated for high‐grade tumours, both groups had deficits affecting forward thinking, inhibition and mental flexibility.^40^

Although BRIEF did not show any significant results in the different indices and no participant had results above the cut off for executive difficulties, 54% of the adults reported difficulties with planning/organisation, with flexibility, and with initiating activities. Moreover 45% reported difficulties with working memory and 36% with inhibition. This indicates that the adult patients experience executive difficulties to some extent in daily life, which can be revealed if the subscales of BRIEF are analysed.

Results of language tests did not show significant language difficulties; except that BNT and the BeSS subtest inference (which tests the ability to reach a conclusion about something from known facts or evidence) were significantly lower than average. A significantly low result compared with normative data in BNT for children treated for infratentorial tumours was also reported by Aarsen.^21^ In addition, the lowest mean results in the intelligence tests were the tests measuring verbal comprehension, which is an indication that the patients had some language difficulties.

The children who performed the academic tests scored mean results in all tests, except for reading speed, which was significantly lower compared with norms. To be a slow reader might affect the child's ability to follow tuition in school and to participate in the often rapid conversation among peers.

Eight patients reported learning difficulties in the interviews. Among these we found the lowest test results, which stress the importance of performing cognitive tests in order to identify patients with potential learning difficulties. In a study by Lönnerblad,^41^ patients treated for brain tumours in childhood performed worse in Swedish, mathematics, and English compared with controls. Interestingly, there were no differences in outcome between survivors treated for high‐or‐low‐grade tumours. This strengthens our findings of cognitive difficulties in some of the investigated patients. This pattern of function loss may be due to lesions to cerebro‐cerebellar circuits following treatment of cerebellar tumours.^7^

We found no correlations between age at diagnosis and the results of the intelligence tests. This is in accordance with a study by Pletschko.^22^ However, we found that age at diagnosis and reading speed were significantly correlated. This suggests that young children with an immature brain are more susceptible to damage, which may lead to learning difficulties.^41^

There are contradictory results in the literature concerning the association between tumour location in the cerebellum and neurocognitive outcome.^37^ In a study by Khajuria,^37^ there was no significant association between tumour location and neurocognitive outcome, which was also reported by Beebe.^19^ Due to our small sample, we could not investigate this.

Patients treated for pilocytic astrocytoma in the posterior fossa have a favourable clinical outcome. However, 40% of the participants in our study have reported considerable learning difficulties that could have been detected by cognitive and academic testing. These learning difficulties were not dealt with in school, and further academic support would have been warranted.

No patient fulfilled all criteria for CCAS, although there are indications that some of the patients had impaired executive and language functions. Some also had problems in visuospatial functions. None of the patients suffered from pCMS.

The limitation of this study is its small number of patients and the cross‐sectional design which makes it impossible to perform investigations to link observed cognitive difficulties with tumour size and anatomical location in the posterior fossa.

The strength of the study is that it was possible to identify all 27 children and young adults diagnosed with low‐grade astrocytoma in the posterior fossa during childhood in a tertiary referral centre during a defined time period and to follow‐up 74% of them. This entails a low risk for selection bias. Another strength of the study is the long follow‐up period (mean 12.4 years; range 5–19 years), which gives a possibility to get a picture of the patients' development over time. However, the long time frame can also be a limitation when comparisons are made between patients at different times after treatment.

## 6 CONCLUSIONS

The long‐term functional outcome for children treated for low‐grade astrocytoma in the posterior fossa is favourable. However, some patients have cognitive and academic difficulties which can be revealed by neuro‐cognitive and academic testing, including interviews of academic performance in school. Therefore, it is imperative to identify those in need of more thorough cognitive follow‐up programmes, including interventions in school. Moreover, there is a need to establish appropriate collaboration between paediatric neuro‐oncology clinics and the educational system.

**Nomenclature**
BeSS: Bedömning av Subtila Språkstörningar (Assessment of subtle language difficulties)
BNT: Boston Naming Test
BRI: Behavioural Regulation Index
BRIEF: Behaviour Rating Inventory of Executive Function
CCAS: Cerebellar Cognitive Affective Syndrome
CELF‐IV: Clinical Evaluation of Language Fundamentals ‐ Fourth Edition
CNS: Central Nervous System
D‐KEFS: Delis‐Kaplan Executive Function System
DLS: Diagnostic Manual for analysis of reading and writing skills
FSIQ: Full Scale Intelligence Quotient
GCS: Global Executive Composite Score
IQ: Intelligence Quotient
LGG: Low Grade Glioma
M: Mean
MI: Metacognition Index
pCMS: Post‐operative paediatric cerebellar mutism syndrome
RCT: Rey Complex Figure Test
SD: Standard Deviation
TMT: Trail Making Test
WAIS: Wechsler Adult Intelligence Scale
WISC: Wechsler Intelligence Scale for Children

## CONFLICT OF INTEREST

The authors have stated explicitly that there are no conflicts of interest in connection with this article.

## AUTHORS' CONTRIBUTIONS

All authors had full access to the data in the study and take responsibility for the integrity of the data and the accuracy of the data analysis. *Conceptualization, Data curation, Formal analysis, Funding acquisition, Investigation, Methodology, Project administration, Resources, Software, Supervision, Validation, Visualization, Writing original draft, Writing review editing*, I.K.; *Conceptualization, Data curation, Formal analysis, Funding acquisition, Investigation, Methodology, Project administration, Resources, Visualization, Writing original draft, Writing review editing*, C.E.; *Conceptualization, Data curation, Formal analysis, Funding acquisition, Investigation, Methodology, Project administration, Resources, Validation, Visualization, Writing original draft, Writing review editing*, M.S.; *Conceptualization, Data curation, Formal analysis, Funding acquisition, Investigation, Methodology, Project administration, Resources, Software, Supervision, Validation, Visualization, Writing original draft, Writing review editing*, B.S.; *Conceptualization, Data curation, Formal analysis, Funding acquisition, Investigation, Methodology, Project administration, Resources, Validation, Visualization, Writing original draft, Writing review editing*, M.T.; *Conceptualization, Data curation, Formal analysis, Funding acquisition, Investigation, Methodology, Project administration, Resources, Software, Supervision, Validation, Visualization, Writing original draft, Writing review editing*, P.F.

## ETHICAL STATEMENT

The study was approved by the Regional Ethical Board (EPN Uppsala Log. No. 2015/107). Informed consent was obtained from all participants included in the study.

## Data Availability

The data that support the findings of this study are available on request from the corresponding author. The data are not publicly available due to privacy or ethical restrictions.
